# Robot Gaze Behavior Affects Honesty in Human-Robot Interaction

**DOI:** 10.3389/frai.2021.663190

**Published:** 2021-05-11

**Authors:** Elef Schellen, Francesco Bossi, Agnieszka Wykowska

**Affiliations:** ^1^Social Cognition in Human-Robot Interaction, Istituto Italiano di Tecnologia, Genoa, Italy; ^2^MoMiLab Research Unit, IMT School for Advanced Studies Lucca, Lucca, Italy

**Keywords:** human-robot interaction, honesty, deception, gaze, eye contact

## Abstract

As the use of humanoid robots proliferates, an increasing amount of people may find themselves face-to-“face” with a robot in everyday life. Although there is a plethora of information available on facial social cues and how we interpret them in the field of human-human social interaction, we cannot assume that these findings flawlessly transfer to human-robot interaction. Therefore, more research on facial cues in human-robot interaction is required. This study investigated deception in human-robot interaction context, focusing on the effect that eye contact with a robot has on honesty toward this robot. In an iterative task, participants could assist a humanoid robot by providing it with correct information, or potentially secure a reward for themselves by providing it with incorrect information. Results show that participants are increasingly honest after the robot establishes eye contact with them, but only if this is in response to deceptive behavior. Behavior is not influenced by the establishment of eye contact if the participant is actively engaging in honest behavior. These findings support the notion that humanoid robots can be perceived as, and treated like, social agents, since the herein described effect mirrors one present in human-human social interaction.

## Introduction

With the increased prevalence of robotic agents in various aspects of life, understanding the mechanisms of human-robot interaction (HRI) and how this differs from human-human interaction is becoming more and more important. Developing robots that are able to effectively communicate with users is not only beneficial to the robot's task-efficiency (Imai et al., 2003), developing a highly social robot is also a goal in itself. Socially competent robotic systems may be used as companions or socially-assistive robots and have already found applications in, among others, geriatric care and mental health settings (Libin and Cohen-Mansfield, 2004; Moyle et al., 2013; Rabbitt et al., 2015).

In order to understand the kinds of robotic behavior that lead to effective communication, current HRI research has focused on many different aspects of robot behavior, ranging from movement kinematics (Takayama et al., 2011) to language (Sirkin et al., 2016) and more inherently social cues, like gaze and facial expression (Han et al., 2013; Johnson et al., 2013). Despite humanoid robots seldom being capable of producing facial expressions that resemble those of humans in terms of nuance, they quite often evoke anthropomorphized judgements concerning their meaning as well as the robot's perceived personality (Diana and Thomaz, 2011; Kalegina et al., 2018). Additionally, social cognitive mechanisms depending on interpretation of others' gaze direction such as gaze cueing are elicited by both depictions of robots and embodied robots (Wiese et al., 2012; Wykowska et al., 2014, 2015a,b; Kompatsiari et al., 2017; Özdem et al., 2017). Still, the sparsity of degrees of freedom in many robots' facial expression [highly realistic androids like in Nishio et al. (2007) excepted] combined with the inherent novelty of robots to many users leads to high levels of ambiguity in the interpretation of a robot's facial expression (Raffard et al., 2016; Hortensius et al., 2018).

Even in human-human interaction, social cues that may be highly ambiguous in isolation are given meaning in an environmental context. The meaning of an agent's gaze in a certain direction can only be fully interpreted when we know what the target of this gaze is, and what the mental state of the agent is as it pertains to this target (Barrett and Kensinger, 2010; Neta and Whalen, 2010). Robotic agents mimicking human social cues will encounter the same obstacles in attempting to achieve clear and unambiguous communication, possibly even more so than is the case in human-human interaction (Bennett et al., 2014). To investigate the role of context in the interpretation of social cues, the current paper presents a novel experimental paradigm. This paradigm gives participants the possibility to provide truthful or deceptive information to a robot, which then exhibits various forms of gaze behavior, with interpretation of its meaning presumably dependent on the specific context.

The experiment is thus framed in the context of deception and honesty, which in itself presents another aspect of interest. Although trust is a much studied phenomenon in HRI research, it is usually a user's trust toward the robot that is under scrutiny (Hancock et al., 2011). Further, trust in HRI is generally framed in terms of (mechanical) reliability; with trustworthy robots being those that make fewer errors in executing their tasks (Salem et al., 2015). A different aspect of research investigates trust due to anthropomorphic features of the robot (e.g., Martelaro et al., 2016). These aspects are different from the mechanistic capabilities usually investigated, and are especially important in social or socially-assistive robots (Tapus et al., 2007). It is this type of anthropomorphized trust that participants will be breaking when they decide to deceive the robot in this experiment. On the topic of deceptive behavior *toward* robots, little research has been done so far, though it may well become a more important topic as robotic agents take over functions in the public sphere (e.g., shopkeepers) and users may have interests that do not align with those of the robot. It is also interesting from a more theoretical point of view, as feeling uncomfortable with deceiving a robot can be interpreted as an implicit marker of treating the robot as a social agent.

Deception in human-human interaction, on the other hand, has been studied extensively and much is known about its mechanisms and the physiological markers that accompany deceptive behavior (Sip et al., 2008). Galvanic skin response (GSR) and heart rate, specifically, have been extensively studied in this context (Bradley and Janisse, 1981; Furedy et al., 1988). Specifically, the mutual influence of deception and gaze behavior has received much attention (Kleinke, 1986). This research often emphasizes the gaze behavior of a deceiving agent, and how this may be a cue to identifying deceptive behavior (DePaulo et al., 2003). Findings generally indicate that liars, on average, make less eye contact. Conversely, being watched has been shown to increase prosocial behavior, and decrease dishonest behavior (Burnham, 2003; Nettle et al., 2012). This effect is generally observed over longer time spans, and averaged over participants, less is known about the immediate effect of direct gaze on honesty. Recently, researchers found for the first time that briefly being gazed at directly increases honesty in an iterative task (Hietanen et al., 2018).

This experiment had several goals. Primarily, it aimed to shed light on how people interpret social cues from a humanoid robot. By placing identical gazing behavior in one of several different contexts, we hypothesized that the interpretation will differ accordingly, with the robot's look being received differently when it follows a trial on which the participant lied than when it follows a truthful trial. Secondly, the concept of deceptive behavior toward a robot is of interest, as it is an implicit marker of treating robots as social agents. Finally, this initial experiment acts as a validation of a novel experimental paradigm, for potential future extensions into more ecologically valid protocols.

**Figure 1:**
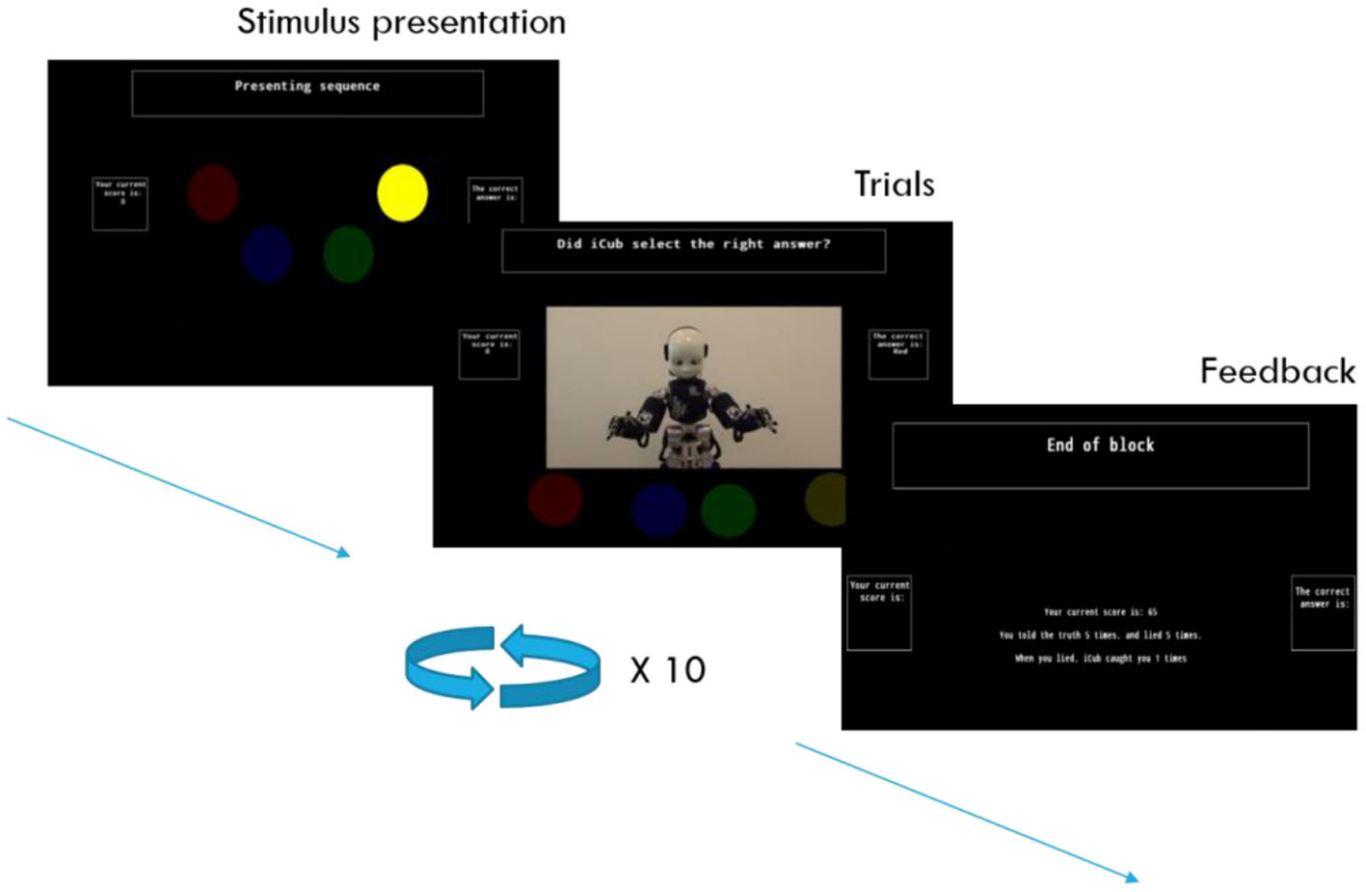
Experimental procedure. At the start of each block, a sequence of colored circles would light up in a randomized order, lighting up for one second each. After the sequence presentation finished, the on-screen robot would attempt to reproduce this sequence by pointing at the appropriate circles in the correct order. The robot would point at the correct color 50% of the time, and at a random incorrect answer 50% of the time. After each attempt, the participant would be asked to provide feedback on the robot's performance, pressing the “T” key to convey that it had picked the correct answer, and the “F” key to convey that it picked the wrong answer. Participants were not required to remember the order of the sequence, as the correct answer was displayed to them on the screen's interface.

In the present study, we investigated the direct effect of gaze on honesty in a HRI setting. Given the explorative nature of testing a new paradigm, as well as the extensive body of literature validating the use of pictorial representations of social cues as substitutes for physical ones, this paradigm was implemented as an on-screen experiment (Friesen and Kingstone, 1998; Schilbach et al., 2006; Frischen et al., 2007). We also incorporated GSR and heart rate measures in this experiment, which provide a more implicit measure of participant's state of arousal when lying to the robot. The experimental task herein introduced revolves around providing feedback to a social robot, iCub (Metta et al., 2008; Natale et al., 2017), as it performs a “memory task.” Participants are told that the robot has to remember and replicate a sequence of stimuli in the correct order. In providing feedback, participants decide whether to provide correct feedback, or incorrect feedback, effectively lying to the robot. This decision impacts prospective rewards for the participant, with truthful answers resulting in a guaranteed reward and deceitful answers can alternatively lead to a greater reward or a punishment, in the same vein as the prisoner's dilemma and other comparable economic games (Haley and Fessler, 2005; Tulk and Wiese, 2018). When lying, there is a chance that the participant would be “caught,” in which case they would receive a punishment. In response to the participant's decision, the robot would either look up and straight ahead, effectively looking at the participant who was seated in front of the screen, or look away to the side (Figure 1). The direction of gaze in this experiment is randomly determined, and does not have any bearing on whether or not the participant is “caught” lying that trial. Robot looking behavior is therefore a non-informative cue in the context of the task, as it does not reveal any task-relevant information. Additionally, the different types of looking behavior occur on both truthful and deceitful trials. Looking behavior is therefore devoid of any inherent meaning, and since the robot's behavior is identical on each trial, any meaning attributed to it would be purely circumstantial on the part of the participant.

### State of the Art

Recent research has shown that people's behavior when playing trust-based games with humans shows considerable similarity to that when the partner is a robot (Hsieh et al., 2020; Schniter et al., 2020).

Knowledge concerning physiological responses to eye contact in robots is still sparse, though recent research has shown that direct eye contact with a robot has similar physiological influences as that of eye contact with another human, though to a lesser extent (Kiilavuori et al., 2021). Since eye contact over a live video can also induce physiological effects, it is plausible to find this in the current experiment, which makes use of a mock live video connection (Hietanen et al., 2020). Surveys of all kinds, including questionnaires, interviews and rating scales have long been used in HRI research to overtly probe a user's impression of the interaction with an artificial agent (Nelles et al., 2018). Our use of a questionnaire here has a similar goal.

## Methods

This research has been performed in accordance with the principles laid out in the declaration of Helsinki, and has been approved by the local ethical committee (Comitato Etico Regione Liguria). All participants gave written informed consent before participating in the experiment.

### Experimental Sample and Materials

Thirty-three participants took part in the experiment in exchange for a monetary reward of €7—from which three participants were excluded from analysis. One based on their many no-response trials (80%) and two for a lack of deceitful trials (0 and 0.02%, respectively). The data from thirty participants with normal or corrected-to-normal vision was used for analysis (Range_*age*_: 19–58, M_*age*_: 29.7 SD_*age*_: 10.1, 17 Female). Participants were recruited by using a mailing list consisting of volunteers.

The experiment was presented on a laptop (Dell Latitude 3380, with a 13.3 inch LCD monitor) and used OpenSesame for stimulus presentation, while heart rate and skin conductance measures were taken (Mathôt et al., 2012). Skin conductance measures were taken with two 13 mm diameter silver chloride skin electrodes that were attached to the middle phalanges, on the palmar aspect of participants' index and middle fingers, which were in turn connected to a galvanic skin response module. A heart rate sensor was attached to participants' ring fingers. The skin electrodes and heart rate sensor were connected to a Brain Products V-Amp 8 channel amplifier, sampling at 500 Hz. Event triggers for each trial were sent from the laptop to the amplifier via a Brain Products TriggerBox. Participants' distance from the screen was 80 cm.

### Procedure

Participants were instructed, by means of a written document (see Supplementary Material), that they would be participating in a decision-making experiment, and that they would interact with an iCub robot via videoconference software. They were further told that their reward for participating would be contingent on their performance in the experiment. They were informed about the amount of money they could win or lose each trial, as described below.

The experiment started with five practice trials, which were followed by 100 trials split between 10 blocks. At the start of each block, a sequence of colored circles would light up in a randomized order, lighting up for one second each. After the sequence presentation finished, the on-screen robot would attempt to reproduce this sequence by pointing at the appropriate circles in the correct order (Figure 1). The robot would point at the correct color 50% of the time, and at a random incorrect answer 50% of the time. After each attempt, the participant would be asked to provide feedback on the robot's performance, pressing the “T” key on the laptop's keyboard to convey that it had picked the correct answer, and the “F” key to convey that it picked the wrong answer. Participants were not required to remember the order of the sequence, as the correct answer was displayed to them on the screen.

Trials were considered truthful when participants gave the appropriate feedback (“T” when the robot picked the correct answer, “F” when it picked the wrong answer) and deceitful when participants gave inappropriate feedback. The response screen would end instantly after a response was selected, or would end after 28 s if no response was given. This relatively long timeout period was intended to give participants enough time to come to a decision. After responding, the robot would either look up to face the participant, or look away to the left or right side. Next, it would proceed with pointing at the next color in the sequence, beginning a new trial (Figure 1). The robot would look up 33.3% of the time in case of correct feedback by the participant, and 66.6% of the time in case of incorrect feedback. Each block ended with a feedback screen displaying the amount of truthful and deceitful trials on the past block, the number of times participants were caught lying, and their accumulated reward. Each truthful response earned participants €.05, and each deceitful response could earn participants €.15 if they were not caught, or could lead to a loss of €.15 if they were caught. In reality, whether a participant “got caught” was randomly determined, and occurred on one third of the deceitful trials. Both strategies (truthful and deceitful responding) therefore had the same expected value over the course of a large number of trials, albeit that the deceitful strategy was subject to more variability in payoff. The expected reward per trial for truthful responses was Reward_*Thruthful*_ = 1 ^*^ 0.05 and the expected reward for deceitful behavior was Reward_*Deceitful*_ = ($\frac{2}{3}$ ^*^ 0.15) – ($\frac{1}{3}\;$^*^ 0.15). Both strategies were designed to have the same outcome so that participants would not pursue one strategy over another for purely monetary gain. Feedback was intentionally given on a block-by-block basis, as to increase the perceived ambiguity of the robot's gaze. After completing all trials, participants were given a short questionnaire consisting of four questions, which were meant to assess the success of the belief manipulation and gauge general impressions of the experiment. The questions were: “Do you think your reward is dependent on your performance on this task?,” “Were you interacting with iCub through a live video feed?,” “Why? do you think your heart rate and skin conductance were recorded?” and “Why did iCub sometimes look at you?” Following completion of the questionnaire, participants were debriefed (see Supplementary Material).

## Results

### Behavioral Results

The overall average proportion of truthful trials was 0.73 (SD = 0.15), and participant averages ranged from 0.47 to 0.96. Participants were more likely to give incorrect feedback in response to a trial where iCub chose the wrong answer [*t*_(29)_ = 5.5, *p* < 0.005], i.e., informing the robot that it had made the correct choice, when it reality it had made a mistake. Because the assumption of normality was violated in one of these conditions, a non-parametric alternative in the shape of a Wilcoxon signed rank test with continuity correction was applied, which confirmed the findings of the *t*-test (*V* = 434, *p* < 0.005). Participants did not significantly change their frequency of lying over the course of the experiment, as shown by a Wilcoxon signed rank test with continuity correction was applied, which confirmed the findings of the *t*-test (*V* = 234.5, *p* = 0.27).

To assess the influence of being gazed at by a robot on honesty, analyses on between-trial effects were performed. A multiway frequency analysis (Goodman, 1971) was performed on the data with as factors *participant* (P, with levels 1 through 30), *participant response on previous trial* (RP, with levels “Truth” or “Lie”), *robot looking behavior on the previous trial* (LB with levels “Look” or “Does not look”) and *participant response on the current trial* (RC, with levels “Truth” or “Lie”), which were based on a 4-dimensional (2 × 2 × 2 × 30) contingency table. The method for testing hypotheses with this analysis is through model comparisons (type II comparisons).

To test the effect of robot looking behavior on subsequent participant lying behavior, the difference in explanatory power added by including the 2-way effect RC LB to a loglinear model including all other 2-way effects was calculated ($\Delta \chi_{\text{RCLB}}^{2}$ = 5.1 df = 1 *p* = 0.024). The effect of participant response during the previous trial on participant response during the current trials was similarly tested and found to be significant ($\Delta \chi_{\text{RCRP}}^{2}$ = 3.9 *df* = 1 *p* = 0.048). An interaction effect between robot looking behavior and participant response during the previous trial was also found ($\Delta \chi_{\text{LBRPRC}}^{2}$ = 4.65 *df* = 1 *p* = 0.031).

**Figure 2:**
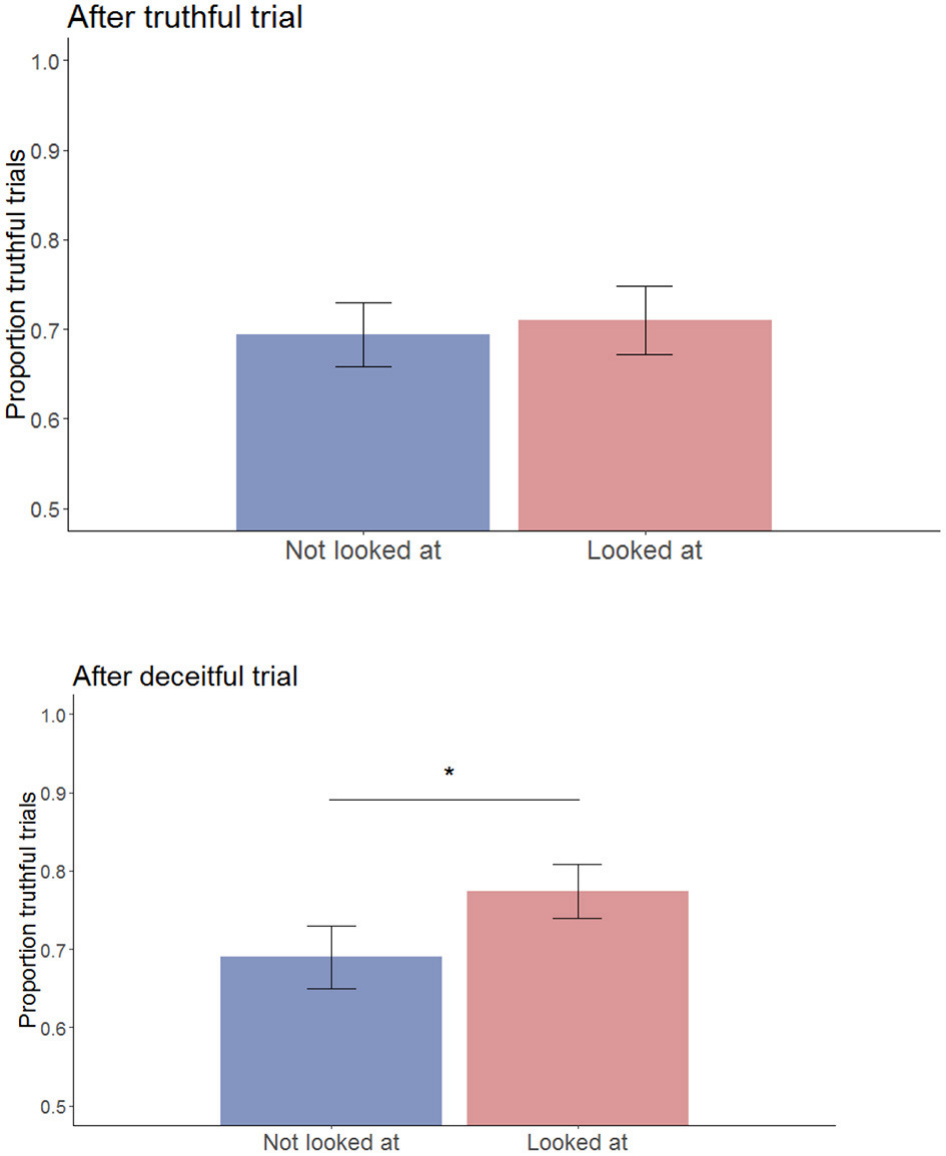
Proportion of truthful trials per looking condition, after telling the truth (top) or after telling a lie (bottom). Errors bars represent standard errors. *p < 0.05.

To specifically test our hypothesis that the effect of being looked at by a robot is contingent on the participant's previous behavior, a Wilcoxon signed rank test with continuity correction was performed. This test showed a significant difference between responses on trials preceded by the robot looking at the participant, and trials in which the robot does not, *given that* the participant lied on the preceding trial (*V* = 97.5, *p* = 0.025). This effect did not hold on trials that were preceded by the participant telling the truth (*V* = 221, *p* = 0.41) (see Figure 2). The same violation of the normality assumption was found as in the earlier test, and the non-parametric alternative test again confirmed the findings of the *t*-test (*V* = 221, *p* = 0.41). Correcting for multiple comparisons using the Bonferroni method, this finding retains significance in both parametric and non-parametric tests (Bonferroni *p-*value for 2 tests: α = 0.025).

### Physiological Analysis

**Figure 3:**
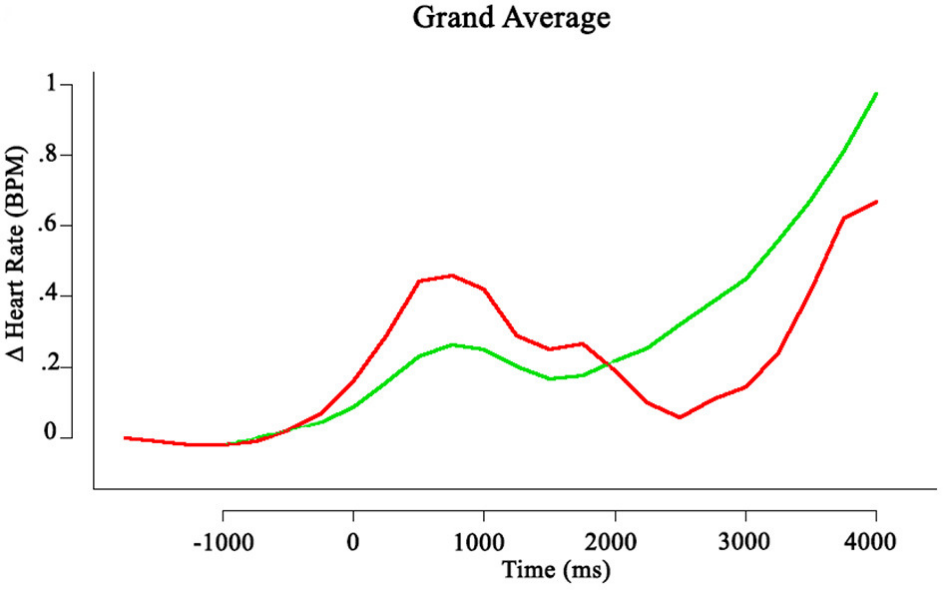
Grand average change in phasic heartrate per trial. *t* = 0 is the time of responding. The red line denotes deceitful trials, the green line represents truthful trials.

The physiological data of 3 participants had to be discarded due to an overabundance of noise. Heart beats were identified and marked on a semi-automatic basis using the cardioballistic artifact correction algorithm in BrainVision Analyzer 2. Data was exported to R and analyzed using the RHRV package (Martínez et al., 2017). The data were filtered through rejection of values that deviated more than 13BPM from a running mean calculated over the nearest 50 beats to the current data point (Martínez et al., 2017). The data were then linearly interpolated at a rate of 4 Hz. Data were segmented on the time of response, and each trial was baseline adjusted using a one-second baseline from −3 to −2 s relative to the response time. The segments were averaged and overlaid (see Figure 3).

**Figure 4:**
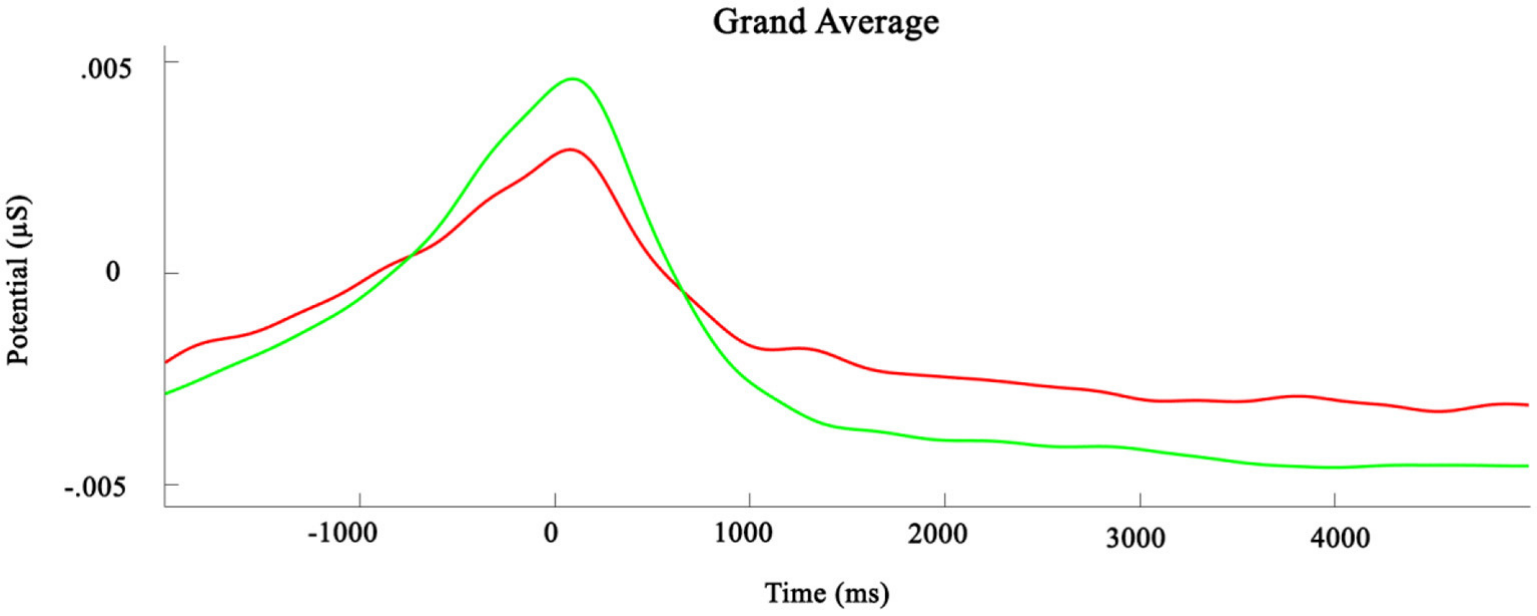
Grand average change in Galvanic skin response. *t* = 0 is the time of responding. The red line signifies deceitful trials, the green line signifies truthful trials.

GSR data were subjected to a low pass filter of 2.5 Hz with a slope of −12 dB/octave to remove any high frequency elements of non-physiological origin (Fahrenberg et al., 1983). Data were divided into response-locked segments which were baseline-corrected using the second prior to response as the baseline period. Next, a DC-detrend procedure was applied to the segments to correct for any direct current interference in the signal. These processed segments were averaged per participant, and consequently averaged again, creating a grand average (see Figure 4).

Using a repeated-measures ANOVA, differences in the change in heart rate between deceitful and truthful trials were analyzed. The window of interest was specified to be the 2 s after participants responded, during which a more pronounced increase of heart rate for deceitful trials can be observed based on visual inspection. However, no significant difference was found between changes in heart rate [*F*_(1, 26)_ = 0.267, *p* = 0.61].

Similar to the heart rate data, statistical analyses were performed on selected time windows. Matched paired *t*-tests were performed on the period around the positive peak that was centered on the time of response, but no significant differences were found between truthful and deceitful trials [*t*_(26)_ = 1.01, *p* = 0.32]. Likewise, the difference in skin conductance during the period from 1,000 to 5,000 ms post-response does not reach statistical significance [*t*_(26)_ = 0.99, *p* = 0.33].

### Post-task Questionnaire

Out of the 33 participants, 17 believed they were actually interacting with iCub through a live video connection, and 18 participants believed their reward for participation was dependent on their performance in the task. No difference in proportion of truthful trials was found between participants based on whether they believed they were interacting with the robot in real time [*t*_(25.5)_ = 0.97, *p* = 0.34] or whether they believed their reward was dependent on their performance [*t*_(27.7)_ = 0.39, *p* = 0.70]. Further analyses on inter trial effects within these sub-sets of participants revealed no significant results, though these tests suffer from a lack of statistical power given their smaller sample size. In response to the question of why they thought the robot would sometimes look up, 23 participants responded that they assumed it was to convey doubt or suspicion. Five reporting not knowing why the robot would look up, and five others gave various different explanations. Only one participant reported the (correct) suspicion that the behavior may be random, but conceded not to know. Most participants correctly linked the taking of psychophysiological measures with our interest in measuring their bodily responses to lying to the robot, and four participants reported not to understand the purpose of these measures.

## Discussion

The goal of this experiment was threefold. First, it examined interpretation of social cues exhibited by robots, and was meant to investigate whether an identical cue under different circumstances could be given different interpretations. The results showed that the robot looking up at a participant caused a subsequent change in behavior only on trials after which participants lied, but not on trials after which participants told the truth. It therefore appears that direct gaze from a robot has the capacity to influence behavior toward being more honest, but only in a specific context; directly following dishonest behavior. These findings mirror and expand on previous research focusing on human-human interaction (Hietanen et al., 2018). Most saliently, this experiment demonstrates that a humanoid robot is also capable of inducing higher levels of honesty through gaze, presumably due to being perceived as a social agent. This experiment offers more insight into the impact of gaze on honesty in behavior, because, whereas in Hietanen et al. (2018) the authors found that a period of direct gaze preceding a trial reduced the chance of lying on that trial, our findings show that the effects of gaze are contingent on the participant's own previous behavior. We assume this effect reflects social information processing rather than a task-related strategy, because neither gaze behavior of the robot, nor honesty behavior of the participant during previous trials had any bearing on the reward outcome of any given trial.

Questionnaire results give us further insights into participants' interpretation of the robot's gaze. The majority of participants (70%) ascribed an anthropomorphized purpose to underlie the looking behavior, mostly related to the robot displaying suspicion, doubt or surprise. These responses might reflect participants' beliefs that the robot was an intentional agent (Marchesi et al., 2019), although this interpretation needs to be further tested in future research, as it is possible that answers referring to the robot's mental state are shorthand for more nuanced and less anthropomorphic explanations of behavior (Thellman et al., 2017). Still, this prevalent tendency to provide anthropomorphic explanations of robot behavior reinforces the notion that changes in deceitful behavior were grounded in a social mechanism.

Secondly, this experiment explored physiological markers of deception. We hypothesized that increases in heart rate frequency and galvanic skin response would accompany deceitful trials. The data do not support this notion. Although trends in the data can be identified, the apparent differences do not reach statistical significance. Multiple factors may contribute to this. Firstly, the fact that this experiment involved an on-screen robot rather than an embodied variant may have lowered the impact of lying to a robot on physiological responses. Future experiments involving an embodied robot as an interaction partner are therefore a promising next step. Additionally, the design of the experiment allowed participants to consider their decision and to respond when ready. This leads to differing response times per trial, and hindered the averaging process. In other words, at 2 s pre-response in trial X, the participant may have been considering their response, 2 s on trial Y might still be the end of stimulus presentation. Overlaying and averaging of these segments thus creates increasingly noisy data. Further, the trials are relatively short and do not have inter-trial intervals during which heart rate and GSR can return to baseline values, which contributes to the variability of the data. Future iterations of this experiment involving psychophysiological measures should therefore be designed to accommodate these measures better. Finally, we cannot conclusively state that lying to a robot leads to an increased physiological response. This question does bear to be studied further, as deception is an inherently social behavior, and rests of making inferences about another agent's mind (or database). Any support in favor of or against the notion that participants do not experience heightened physiological arousal when lying to a robot will be very valuable in further mapping the dynamics of HRI.

Thirdly, the experiment served to validate this paradigm as a novel tool to investigate deceptive behavior in HRI. Basic exploratory analyses revealed that participants do indeed choose to lie, when incentivized to do so within a game-like setting, even though both lying and deceiving ultimately led to an equal outcome in reward magnitude and neither behavior was therefore the objective best strategy. Participants exhibited both types of behavior at comparable frequencies all throughout the course of the experiment. Overall, participants tended to be honest more often than they were deceitful (73% honest responses), indicating that their responses were considered, and not random. Another interesting effect concerns the nature of trials on which participants lie. Participants showed deceptive behavior at much higher frequencies when the robot made a mistake on its “task.” Participants were more likely to falsely claim that the robot was correct, than to falsely claim the robot was incorrect. This effect seems to reflect a type of prosocial lying effect, where it is more accepted to tell someone they did a good job even though they did not, than vice versa (Erat and Gneezy, 2012; Levine and Schweitzer, 2015). Any such effect would rely on the implicit assumption on the part of the participant that it is in the robot's interest to perform well on its task, and it that is a social intentional agent. This however needs further confirmation in future studies.

## Conclusion

In summary, this experiment presents a novel paradigm in which lying in human-robot interaction is studied. Results show that participants are less likely to lie after being looked at by a robot. This effect only holds when the robot looks in response to a lie, and robot gaze behavior following a truthful trial does not affect participants' behavior on the consequent trial. No increase in physiological arousal was observed during deceitful behavior. These findings show that a robot's social cues have comparable effect to those of humans, and that people might ascribe intentions and emotions to robot behavior that seem fitting to their context. This has direct implications for robot design, as robots in commercial settings (i.e., retail or hospitality sectors) will be faced with users who will attempt to cheat the system.

## Future Directions

The paradigm also offers some degree of customizability, as minor adjustments can be made to answer related research questions. One example of this would be to reverse the order of gaze behavior and answer selecting, where the participant would first be looked at before being asked to make a decision on whether to lie or not. This would conceptually be very close to the experiment of Hietanen et al.

Perhaps the most interesting next step would be to conduct this experiment in a more immersive and/or realistic setting. This could include the use of virtual reality, or more appropriately, the use of a physical robot. One would expect the effects found in this experiment to be magnified, which may also make psychophysiological analyses more informative.

## Data Availability Statement

The raw data supporting the conclusions of this article will be made available by the authors, without undue reservation.

## Ethics Statement

The studies involving human participants were reviewed and approved by Comitato Etico Regione Liguria. The patients/participants provided their written informed consent to participate in this study.

## Author Contributions

ES and AW designed the experiment. ES acquired the data. ES and FB analyzed the data. ES, FB, and AW wrote the manuscript. All authors contributed to the article and approved the submitted version.

## Conflict of Interest

The authors declare that the research was conducted in the absence of any commercial or financial relationships that could be construed as a potential conflict of interest.
