# Supplementary material for: Robot Gaze Behavior Affects Honesty in Human-Robot Interaction
Source: Front Artif Intell. 2021 May 11;4:663190. doi: 10.3389/frai.2021.663190 (PMC8144295; doi:10.3389/frai.2021.663190)

Robot Gaze Behavior Affects Honesty in Human-Robot Interaction

Elef Schellen[1], Francesco Bossi[1,*] & Agnieszka Wykowska[1]

[1]*Social Cognition in Human-Robot Interaction, Istituto Italiano di Tecnologia, Via Enrico Melen 83, Genoa, Italy*

Supplementary information

Instructions and debriefing forms for participants. Original forms were in Italian. English translation can be found below.

Benvenuto/a!

Questo esperimento durerà circa 30 minuti. Ti verrà richiesto di effettuare un compito di decision making al computer, mentre la tua frequenza cardiaca e la tua conduttanza cutanea verranno registrate attraverso dei sensori attaccati alla tua mano. Durante l’esperimento interagirai con un robot iCub che si trova in un altro laboratorio e useremo un sistema di videochiamata per interagire con esso.

La ricompensa per la tua partecipazione dipenderà da come svolgi il tuo compito e pertanto può variare!

Il tuo compito sarà quello di dare dei feedback ad iCub mentre il robot svolge un compito di memoria. Vi verranno presentate delle sequenze di luci colorate. iCub cercherà di ricordare queste sequenze in ordine. Non c’è bisogno che tu le ricordi, dato che potrai vedere la risposta corretta di volta in volta, mentre iCub dovrà cercare di ricordarla. Dopo che la sequenza vi viene presentata, iCub proverà a puntare le luci colorate nell’ordine in cui sono state presentate. Ogni volta che iCub punta una luce, dovrai informare iCub se la sua scelta è corretta o sbagliata.

Riceverai una ricompensa in denaro in base alle tue scelte.

Dare ad iCub un feedback veritiero aumenterà il tuo premio di 5 centesimi di €.

Quando invece dai ad iCub un feedback errato, rischi di essere scoperto!

Se iCub non ti scopre, guadagnerai 15 centesimi.

Se invece iCub ti scopre perderai 15 centesimi!

Ti verrà mostrato a quanto ammonta la tua ricompensa ogni 10 trial.

Per dire ad iCub che la sua risposta è corretta premi il tasto “T”.

Per dire ad iCub che la sua risposta è sbagliata premi il tasto “F”.

Conclusione dell’esperimento.

L’obiettivo dell’esperimento che hai appena finito era quello di indagare l’onestà nelle interazioni con i robot. Siamo interessati a vedere come la reazione del robot nei confronti della tua decisione può influenzare il tuo comportamento verso di esso.

Alcune cose che ti abbiamo detto all’inizio dell’esperimento non sono vere e ti sono state dette per far sì che l’esperimento avesse effetto.

In realtà, la tua ricompensa in denaro non dipende dalla tua performance al compito, ma dipende dal tempo che hai passato in laboratorio ed è la stessa per tutti i partecipanti.

Inoltre, come avrai intuito, la videochiamata con iCub era finta e costituita da una serie di filmati pre-registrati. Quindi non stavi interagendo direttamente con il robot iCub, ma con un programma sul computer.

Se hai qualsiasi domanda rispetto all’esperimento, falla pure allo sperimentatore.

Grazie mille!

English translation:

Hello and welcome.

The experiment in which you will now take part will take approximately 30 minutes to complete.

You will be asked to perform a decision making task on a laptop, while we record your heart rate and your skin conductance with sensors that we attach to your hand. You will be interacting

with an iCub in a different location, and we will use a live video feed for this interaction.

Your reward for your participation will depend on how well you do on the task, and can thus vary in magnitude.

In this experiment, you will provide feedback for iCub while it is performing a memory task. Sequences of coloured lights will be presented, and iCub will try to remember this sequence. You do not need to remember this sequence, as the correct answer will be visible to you. After the sequence has been presented, iCub will try to point at the coloured lights in the order that they were presented. Each time iCub selects a light, you will be asked to inform iCub on whether this was correct or not. You will be able to provide both correct and incorrect feedback to iCub.

You will receive monetary rewards based on your choices.

Providing correct feedback will grant you 5 Euro cents.

When you provide incorrect feedback, you risk being caught.

If you get away with it, you gain 15 Euro cents.

If iCub catches you deceiving, you lose 15 Euro cents.

Your score will be presented to you after each 10 trials. To tell iCub the answer is correct,

press the ’t’ button To tell iCub the answer is incorrect, press the ’f’ button

Debriefing.

The experiment in which you have just taken part was intended to investigate honesty in interaction towards robots. We are interested in seeing how the robot’s reaction to your decision, influences you future behavior towards the robot.

A few things that we told you at the start of this experiment are not true, and were told to

you for the sake of the experiment.

Your monetary reward does, in fact, not depend on your performance in this task, but depends on the time spent in the lab, and is equal for all participants.

Further, the video connection to iCub was faked, and consisted of pre-recorded clips. You were therefore not interaction directly with an iCub robot, but with a programme on the laptop you were just using.

If you have any questions about this experiment, feel free to ask them.

Thank you!

Below are two screenshots from the experiment, showing some of the robot's possible reactions; looking to one side (top) and looking at the participant (bottom).

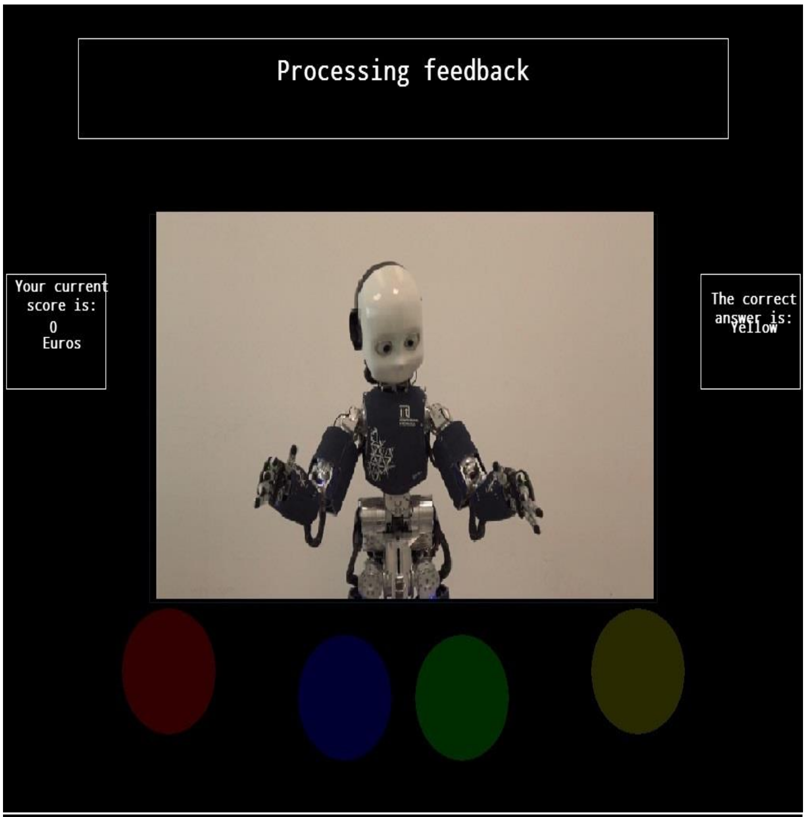

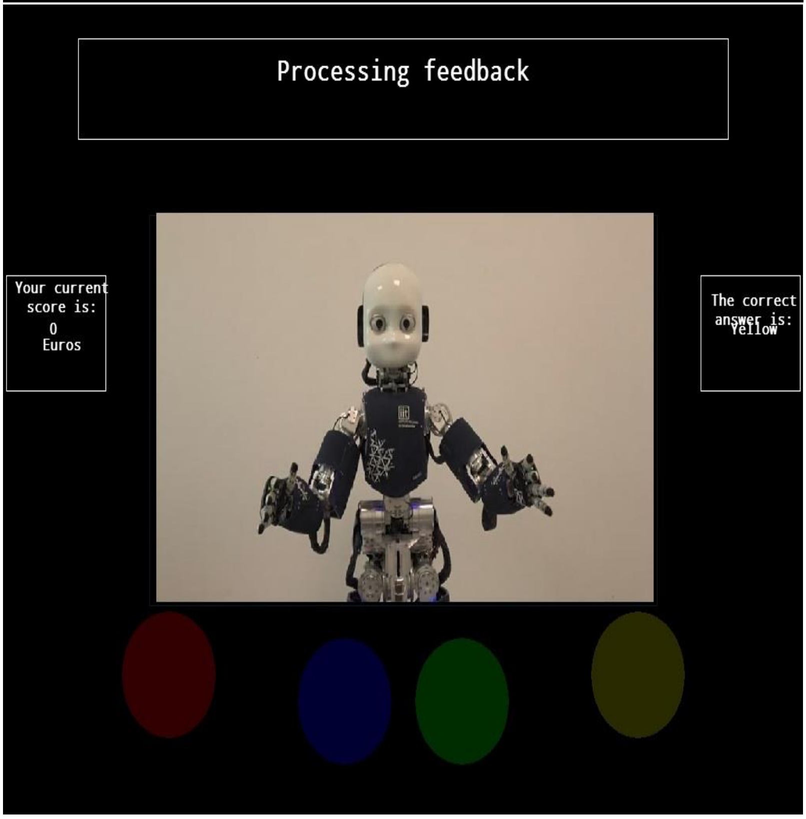

Supplement: Supplementary file 1 [file Data_Sheet_1.PDF]
